# The De Lesseps Canal in Its Relation to Hygiene

**Published:** 1880-07

**Authors:** G. Halsted Boyland

**Affiliations:** Baltimore, Md.; Late Surgeon French Army, Diploma of the International Convention, Bronze Cross of Geneva; Author Six Months under the Red Cross with the French Army; Of Contributions to Various Scientific Journals, etc.; Member of the Medical and Chirurgical Faculty, State of Maryland; Corresponding Member Cincinnati Academy of Medicine, etc., etc.


### The Practitioner.

Vol. I.——*July, 1880.*——No. 7.

ARTICLE I.

### THE DE LESSEPS CANAL IN ITS RELATION TO HYGIENE.

BY G. HALSTED BOYLAND, M. A., M. D., ETC., BALTIMORE, MD.

Late Surgeon French Army, Diploma of the International Convention, Bronze Cross of Geneva; Author Six Months under the Red Cross with the French Army; Of Contributions to Various Scientific Journals, etc.; Member of the Medical and Chirurgical Faculty, State of Maryland; Corresponding Member Cincinnati Academy of Medicine, etc., etc.

The vast importance of the inter-oceanic canal as proposed by M. De Lesseps, to the commerce of the world, but more especially to that of the United States of America, is sufficiently evident to all clear thinking men.

It is not the purpose of this paper to discuss the different routes suggested for inter-oceanic transit, it being assumed at the outset that a sea level canal, without locks and the shortest is more desirable than a long one with such obstacles. But when in addition to the advantages of time saved and distance gained—two essential points to trading vessels—a whole country is rendered more salubrious, a people more healthy, and the means of transmitting the germs of infectious diseases to foreign ports materially diminished, such a canal assumes an interest more vital than that of gain and the need of its construction, becomes apparent upon grounds higher and farther reaching than purely commercial ones. The ratio of prevalency of infectious diseases decreasing as the latitudes increase north, the Panama canal built through the most malarious country of the western hemisphere, would naturally be productive of more hygienic benefit both by land and by sea, than one built further north. Conducting large bodies of water through marshy districts, acts directly upon the districts themselves, and brings about also, certain atmospheric chemical changes. The numberless stagnate pools, sluggish streams and morasses, lying in the direct line along which the canal will be built, will be opened up and drained of their miasmatic infectious properties, whilst the motion of the water in the canal will not only exert a cooling influence upon the atmosphere, but vapors arising from its surface will neutralize those malarial mists so characteristic to the tropics. The sanitary benefits derived will then be two-fold: 1st. Those affecting the region itself—land. 2d. Those affecting foreign intercourse—maritime.

It is only necessary to consider the principle upon which the earth is warmed and that upon which atmospheric moisture is developed, to see how water in nature is held in a perfectly round and ever returning circulation. As it falls from the clouds, flows from mountain springs and meadows, the rivers emptying themselves into the ocean—so it rises in the form of vapor from the surface of the ocean and other bodies of water as well as from moist ground to the clouds and falls again from here upon the earth, its elevation being caused by the sun. Where the influence of the sun is greatest, this circulation is carried on with the most activity. It is important to an understanding of this motion of water, to remark the facility it possesses for warmth and its tension, that is, its inclination to change into vapor; because under the influence of heat the water not only plays a passive role but there is also an inner action developed that direct observation cannot discover; so that the exact relations of water in nature cannot be appreciated quite rightly. The surface of land which the northern hemisphere embraces, is three times as great as its extent on the southern hemisphere, and also much richer in rivers. As therefore, on the northern hemisphere especially, the precipitate must be greater than the amount of vaporized water, so it follows, that the contrary holds good on the southern hemisphere, where the quantity of vaporized water exceeds the condensed. No where on the earth's surface can these phenomena be better studied than on the paludal isthmus of Darien, a neck of land so fitted by nature for them. The American Cordillera is here broken into detached mountains which sink in attitude until the lowest elevation is attained as they approach Chagres on the Atlantic and Chorera on the Pacific side. There is a succession of very wet lands or marshes separated only here and there by these hills which farther south rise into the

Andes. The highland is heavily wooded, the lowland being covered with all kinds of tropical vegetation and undergrowth, which coupled with the quantity of water often stagnant in small bays, inlets, pools, etc., only require the rays of a southern sun to make them exhale that miasma so fatal to man.

Such is the general physical geographical character of the land through which M. De Lesseps will build his canal, utilizing in part, the bed of the Chagres. This district, it is proposed to open up and cleanse also by means of channels conducting to the Atlantic, in order to obtain drainage and perfect security to the dam at Gamboa, between Cruces and Matachin. Similar channels will be constructed on both sides of the canal for the drainage of smaller streams. The Chagres, Obispo and Trinidad rivers will be drained in the same manner. These influences as well as the atmospheric ones, will undoubtedly be beneficial not only across the whole area of the isthmus, but also, will extend many miles in every direction, reaching the vicinity of Porto Bello, one of the hottest and most unhealthy known. The construction of the canal and the channels to regulate the flow of the rivers and streams will thus effectually wash out and cleanse beyond any possibility of return, these marshes, keeping up a system of drainage and atmospheric process, which will tend to lower the high temperature and render the whole region of the isthmus more salubrious, thereby reducing the causes of putrid and miasmatic emanations, the direct agents in the production of infectious diseases, which flourish in a land where the thermometer even during the rainy season, seldom falls below 87° Fahrenheit, a temperature considerably above that required for the development of malarial, typho-malarial and yellow fevers. A blow will be struck at the root of the evil; the putrid and miasmatic emanations causing these diseases and rising from the morasses of the isthmus, will be destroyed. Such emanations are generally conceded to be the type of insalubrity. It would be difficult if it were otherwise, for they act as well by their composition as by their inner nature. They strike the senses and produce upon the least delicate organs, a painful and resisting impression, which takes the form of instinctive repugnance and appears, as it were, the forerunner of real danger. It would be exposing one's self to almost certain error to ignore this capital fact in the interpretation of those phenomena which produce putrid emanations. Organic matter both animal and vegetable produces by its decomposition gaseous or volative products which chemistry is able to characterize with certainty. They furnish principles placed rightly or wrongly under the comprehensive term miasma, yet quite distinct in their effects as also without doubt, in their native principles, in which seem to be the essential properties, and so to speak, secret virtue of these emanations. The gases arising from animal and vegetable decomposition are all in different degrees unfit for respiration or poisonous.

The putrid emanations although always present on the isthmus vary more or less in direct proportion to the amount of decomposing animal matter; contributing their quantum to the fons et origo morbi; they nevertheless, hold an ætiological position quite insignificant, as compared with that of the miasma of vegetable decomposition, pervading the low and swampy ground east and west of the Culebra or elevated section, as designated by the report ol the International Technical Commission, appointed to examine the definite work required for the construction of the Panama canal; signed by messrs. Totten, Dirks, Boutan, Wright, Dauzats, Sosa, Ortega, Couveux and Blanchet. It will be observed that these still pools, small patches of water, and turbid streams, are caused by the overflow of the sea and rivers, with which the Isthmus of Darien is so well provided, mostly, however, by the latter, that is by fresh in chemical distinction from salt water, to which, nevertheless, a few such owe their existence not far from Colon and Panama, and although of little consequence as regards number in comparison with the many fresh marshes, yet by their mixture with them they make these all the more deleterious. The land in these districts like that in most malarious ones is little cultivated, abounding in alluvial soil, alternating with highland thickly wooded with storax, caoutchouc, various dyeing drugs and other tropical trees.

Beyond the products of decomposition that chemistry discovers, putrid emanations demonstrate the existence of a particular principle entering rather into the laws of organic nature and that their effects approach those of organic poisons. This was recognized by Berzelius, and it is curious to observe this former hypothesis become a certainty confirmed by procedures in the practice of disinfection. Putrid vapors do not differ from deleterious gases unfit for respiration; nevertheless, independent of their nature and modes of action, there exists relative to the effects that they produce upon the life of animals as well as man, a sort of contradiction; only apparent, however, when it is taken into consideration that the same poisonous emanations will produce harmful results, differing in their outward expression in correspondence with the diverse temperaments exposed to their influences. There should, therefore, be no confusion in hygiene, which in the instance in question, will remove the cause of the evil by drainage. The most important point of view with reference to poisonous emanations, is the extension of their effects upon great masses of men who unwillingly and oft unwittingly, find themselves exposed to them. There is no lack of statistics upon this subject, and their authenticity and gravity give them a really scientific value.

Pestilential diseases of which ancient history has handed down the accounts, have almost invariably been explained by the insalubrity which makes districts like these, centres of corruption. Thucydides, Diodorus, Titus Livius, in their immortal writings, have pictured these sources of death in the most vivid colors. Galen, among the causes which he assigns to pestilential fevers, signalizes the putrid condition of the atmospheric air occasioned by decomposing animal matter. St. Augustin reports that a great quantity of grasshoppers drowned in the sea and washed up on the coast, where they putrified, became the cause of a most cruel plague. In modern times Ambroise Pare saw on the sea coast of Tuscany and at Venice, a pestilential fever which made great ravages, and was traceable directly to a kind of small fishes which had putrified in great number in that part of the Adriatic. M. Tardieu in his admirable work on public hygiene, indicates strongly and almost at every step, the pernicious effects of putrefaction of animal substances. It is useless to prolong details that can only give an imperfect idea of the reality. Such animal matter exposed to the combined action of humidity and a tropical sun, develops the mephitic gases belonging to that climate. The bad odor would of itself be a sufficient ground for hygienic interference with much more reason than when the public health is imperilled.

To undertake hygienic measures in a vast area like this would be almost a hopeless task and would require in addition the expenditure of millions on the part of the Colombian government. M. De Lesseps undertakes these for them, to the profit of foreign nations also.

The vegetable effluvia from these morasses and miasmatic rivers of which the Chagres, Trinidad and Obispo, with the smaller streams belonging to them, are excellent examples, constitute by far the greater and more formidable causes of malignant infectious fevers.

Under the name marsh being comprehended, every portion of soil alternatively covered with and abandoned by water and giving rise under the influence of drying and heat, to those miasma that engender fevers, ponds, inlets, overflow of rivers or sea, flat exposed shores, mouths of rivers, stagnant pools, torpid and sluggish streams, etc., which abound on the isthmus of Darien; such become with equal facility and under conditions the most varied, the centres from which the miasmatic poisons of decomposing vegetable matter emanate to alter or destroy the health and constitution of individuals, or often of a whole people.

If it were possible to take in the whole surface of the globe at a single coup d'oeil, it would be seen that although there are large portions of soil occupied by stagnating waters in all countries, yet no where is this more prevalent in proportion to the number of square miles, than on the isthmus, the great swamps of the southern states of North America not excepted. There are here three classes of marshes to be dealt with, viz.: those formed at a very moderate elevation by ponds and large swampy valleys, with a slant so unimportant that the flow of water from them is very feeble; those formed by the beds of rivers and streams transformed into morasses by the dryness and heat of the tropics; and those formed by the patches of salt water on both the east and west littoral. These consist at times, of larger or smaller low paludal planes of brackish water, divided up again into smaller ones; at others, of salt meadows unequally submerged and connected by rivulets of water having sandy borders. By reason of the general disposition of these salt marshes to mingle with the fresh ones and contaminate them still further, they demand cleansing in the same manner, and will also be swept out by the dredging along the line of the canal. They are to be classed as artificial in distinction from the natural turbid and simple ones farther inland, where the alluvial, little permeable, argillous and argillo-silicious soil owes its abundant supply of stagnating water less to torpid springs and rainfall than to the rivers and streams. These more or less miry and of an odor and taste often fetid, nourish a special vegetation as well as a mass of infusoria and vibrios. From their surface arise incessantly but more actively during the night gases of phosphoric or carbonic hydrogen and carbonic acid. Under the influence of light even quite diffuse and of the green animalculæ spread with profusion upon them, these waters acquire a degree of oxygenation that can go as high as 61 p. 100 of dissolved air; from which result variations in the proportion of oxygen that they discharge into the atmosphere, as well as of carbonic acid; which under the same influence, is partly decomposed. It is permitted to appreciate rather than demonstrate the relation of this double phenomenon to miasmatic effluvia. A fact that can be more readily seized but none the less essential, is the formation of sulphuretted hydrogen, resulting from the decomposition of the sulphates by organic matter in these stagnant groups of salt and fresh water. The destruction of aquatic animals and vegetables for whom this mixture is deadly, is still another and patent cause of insalubrity; they thus become a source of pestilence in and per se, apart from the jeopardy in which they place public health. It is not only, therefore, in the neighborhood of the sea, and at the mouth of the rivers but throughout the whole isthmus in districts far from the *littoral*, that the lands by reason of their composition give rise to these reactions, as has been shown by the savans Salvi and Saddi. The quality of the waters, the composition of the soil, the presence of organic matter, constitute principally the elements of marshy emanations. Whatever the classes of causes may be either isolated or united, they cannot suffice to reveal the nature of these deleterious miasmata that engender fever of those effluvia of which it may be truly said "by their fruits ye shall know them." They remain ignored by their very essence, and can only be designated under the mysterious and poetical name, malaria. However incomplete, despite the efforts of science, our knowledge on this subject may remain; there is a field sufficient for study and one fruitful enough as regards practical medicine and public hygiene, if indeed it is desirable to search out the conditions that produce and send off miasma, their mode of action and what is above all important in this consideration, the means of destroying the hot beds where these exotics flourish. The principal factors influencing the production of effluvia are the state of the surface of evaporation and elevation of temperature. The temperature on the isthmus of Darien being quite elevated at all seasons of the year the morasses are continually active to a greater or less degree in exhaling miasma—especially so immediately after the rainy season, during the months of December and January. The state of these marshes being neither one of complete dryness nor one of complete submersion at any time, is a most excellent one for giving off effluvia from their surface and spreading the germs of fevers of the intermittent, typho-malarial, malignant typhoid and yellow fever types. The effluvia carried by atmospheric vapors are favored by the influence of the sun, falling in the evening and during the night, in a measure that corresponds to their condensation. It is at that time that their deleterious action is most to be feared. Their dispersion does not always take place in an equal manner and although they pervade the whole isthmus generally, yet in certain valleys and depressions the malaria seems to hover and concentrate; this is particularly the case on the Atlantic side a few miles from the bay of Limon, and on the Pacific near the mouth of the Rio Grande.

Puvis, in his valuable researches on stagnant waters has calculated that where they occupy only a two-hundreth part of the soil the pernicious effects of miasmata make themselves felt upon a thirteenth part of the whole country. What then must be the result when the general character of a given tract of land is wet and marshy? Dr. Lefevre assures that the swamps du Brouage send their effluvia as far as Rochefort, distant 7 or 8 kilometres. It is known that effluvia follows currents of air, and winds, and as the infection can be carried miles in this manner—as witness the fact that wind blowing from the east across Holland, brought the fever to the coast of England, so the air purged of malaria and rendered respirable would make its beneficial influence felt to a distance at least equally as great. M. Melier has also observed this in the cities and towns situated at some distance from the Marennes marshes. The limit of height which miasmata attain is much more restrained. The different stages of elevation suffice on the Campagna Romana to at first attenuate and finally overcome their action; there is no doubt about the height to which the purifying qualities of the salt water vapors from the surface of the inter-oceanic canal would reach their health-giving properties would, therefore, as far as that is concerned, be much more than sufficient to overcome the septic tendency of malaria. Granting that malarious exhalations lose their nocent power by elevation, it is not until they have spent themselves upon the regions round about at the expense of public health and life. It is not designed to trace the effects produced by malaria upon living beings, but rather to recall in this place a few statistics given by distinguished observers with reference to the cleansing of marshes such as those on the isthmus of Darien by the introduction of canals and channels—the only means of thoroughly eradicating them.

The vegetation owing to the soil, presents a character quite peculiar. Aquatic plants grow with great vigor; the trees are mostly of a stunted and mean growth, it being difficult to bring their fruits to maturity; cocoas and bananas perhaps, excepted. In general, they remain gorged with watery sweetish sap, tasteless and without aroma. The cereals about Panama, where the land is to some extent cultivated, are of a very inferior quality, and pot plants succeed but imperfectly. The legumens are cold and abound in watery principles.

The trees and plants for this reason take very little part in rendering healthy such marshes by absorption of their noxious gases; being rather kept down under their influence and weakened by the enormity of the task. But it is, above all, the animals next to man that suffer most. This fact did not escape the ancients and nobody is ignorant of the counsel given by Vitruvius to examine the intestines of sheep to ascertain the salubrity of certain places. The animals met with on the marshy lands of Central America are generally small, thin, with little activity and subject at certain periods to fatal ravages of the epizootic. It is the same with man.

The inhabitants of the isthmus, owing to intermingling of races, occupy comparatively an inferior position in the social scale, whilst the bad influence of a marshy district finds expression in their characteristic physiognomy, which carries upon it the marks of the conditions in which they live. Without speaking of their miserable surroundings as a class, their constitutions are from the time of their birth greatly altered by a cachexia peculiar to them and to their small stature. They have a wan, sallow tint, with softness and puffiness of the tissues, poverty of the blood, extra large development of the stomach, congestion of the liver and spleen, tendency to hydrosis. This is accompanied by a state of languor, dullness of the intelligence and of the entire nervous system. Such is the picture of individuals exposed to this malaria, which manifests itself by the slow alteration of the sources of life and by engendering fevers the divers forms of which have been attributed to the different degrees of its energy, varying in most countries according to the nature of the waters, the state of the marshes, the season and climate; but which in these latitudes remains more fixed. And if man in some exceptional instances, by reason of his strength and constitution is able to resist malaria, he can never accustom his nature to its influence. There is no acclimatization possible. It must not be forgotten that the cause of individual disease is not the only one which claims attention; the question is higher and broader, it being upon a whole population that the malaria makes itself felt; moveover the average of human life is much lowered as abundant statistics prove. M. Becquerel found that the average of human life in marshy districts was reduced to two-thirds of that in dry and more elevated ones; and that it had often happened in such localities in France, that of all the young men conscripted not one was fit for military duty. Further that there were years in which there remained not one of the class called; all had died before the age of conscription and most in infancy. M. Lefevre assures that the fact of a whole population reduced to nought had recurred several times during his administration near malarial centres. In the report by M. Melier of his work in public hygiene are given data in which the mortality in some instances reaches as high as 1.13 of inhabitants. All observers agree that although it is principally upon new born babes that this excessive mortality prevails, yet these miasmata, none the less, reduce in a very great degree the average duration of life in the adult population. The rates of disease and death being in proportion to the extent of marshes and malarial conditions generally, and to the number of individuals coming under their influence, this average is higher in the United States, England, Holland, France and Italy, than in equatorial latitudes. The effluvia of marshes not only exercise their pestilential power upon the inhabitants of Central America, but send it abroad by passengers, baggage, bales, packages, etc., and by ships; not only are their towns and villages often decimated, and the average of human life reduced to a very low one, but the far wider reaching danger is incurred of spreading infectious diseases to foreign ports, be the quarantine regulations never so strict.

For such evils nothing should be neglected to find a remedy. The combined efforts of power and science are barely at the height of the mission; and on principle there is not a government in the world worthy the name, that would not, as a point of honor, be ready to show its solicitude for these grave problems, which interest so directly the public health, and to give impulsion to the grand work that alone can destroy the centres of infection constituted by the morasses of the Darien Isthmus. The means usually employed to combat malaria, belong to hygiene, it is true, but it is readily understood can only be palliative and feeble in face of the extensive tract of swampy lands that will be encountered along the line of the canal. The disposition of habitations exposed to miasma, the importance of proper clothing and strengthening moisture, precautions with reference to regulating hours of work, and the duration of working hours, the efficacy of tobacco as a prophylactic, of the salts and preparations of quinine do not reach the origin of disease, and all, it must be confessed, fall to the ground before the want of means and absolute destitution of most of the inhabitants scattered along from Colon to Panama. It is to more radical measures that we must look for destruction of the miasmatic pestilence; measures all the more interesting because they belong to the domain of science, which as regards hygiene, should stop at nothing that may contribute to the protection of public health. The end to obtain is precise. To obviate the alternate states of dryness and inundation prevailing upon the isthmus, and to avoid stagnation of water. The indication is fulfilled by draining and causing to flow off accumulated waters and by cleansing the morasses of the plains and valleys. Even this would leave matters better only until the next flood or rainy season, when there would be a return of the old trouble were it not that the dredging will go to a depth sufficient to guarantee against it, which with the channels provided on both sides of the canal, as well as the canal itself, will make such a recurrence an impossibility. The power of infection greatest on the surface of marshes, decreases regularly with the depth. It will be seen by again referring to the report of the Technical Commission, that the dredging will reach a point beyond that at which malarious infection will have ceased to exist; some approximation may also be made to the amount of water to be conducted through the Panama canal. The dimensions of the wet section of the canal which the Commission has adopted are as follows: 1st. Between Colon and Kilometer 36 (the Atlantic Division,) and between Kilometer 61 and Panama, (the Pacific Division.)

| | |
|---|---|
| Width at bottom, - - - | 22 Metres. |
| Width at water line, - - - - | 50 Metres. |
| Depth " " - - - | 8.50 Metres. |

2d. Between Kilometer 36 and 61, (the Culebra or summit division.)

| | |
|---|---|
| Width at bottom, - - - - | 24 Metres. |
| Width at water line, - - - - | 28 Metres. |
| Depth " " - - - | 9 Metres. |

II.

The establishment of canals and irrigation in different countries, has invariably been attended with most beneficial effects as to public hygiene. The city of Leipzig which had been afflicted by epidemics of Asiatic cholera, has enjoyed entire immunity from them of late years. In fact, there has not occurred a single epidemic since the establishment of the canal through the city. Drainage from the surface of marshes would be useless, owing to the continual filling in from the sides of streams that might be provided for that purpose, and to their great tendency to stagnate; and in such conditions subterraneous channels would be out of the question. The only thorough plan is the introduction of deep dredging machines, so skilfully utilized by the Dutch. In the marshes of Tuscany after making fruitless attempts to dry them up by means of evacuating the water, carried out by the Princess of Tuscany and the Austrian Dynasty, the Grand Duke following in the footsteps of his predecessors, adopted the system proposed by Lacuee to Napoleon, which consisted principally in turning into a canal the waters of the Ombrone and several adjacent streams conducting it through, sweeping out and cleansing the morasses, which were first dredged; the canal being given issue in the direction of the sea. These Tuscan marshes like those on the isthmus of Darien were composed of salt and fresh ones, a mixture acknowledged to be more prejudicial to public health than either by themselves.

The results obtained were most gratifying, and the success complete. The marshes were then cleansed and the causes of infectious fevers removed to the great benefit of public health, thanks to the munificence of that Prince, who spent 12 million francs of his personal fortune in this noble work. There are numerous other instances upon record in which the construction of canals through marshy districts have been productive of the greatest benefit to public health, have eradicated the evil, and guaranteed against a return of it. The principle, then, remains fixed. The construction of the De Lesseps canal will only be another verification of it on a gigantic scale. From a hygienic standpoint also, a sea level canal and without locks is most desirable, the only other example in the world being its brother and complement the Suez canal, whose sanitary influence upon the surrounding country is borne testimony to by the general immunity from disease. Thus the means most sure and most direct of cleansing and eradicating marshes and of causing to disappear the diseases peculiar to them is the construction of canals and drainage channels; such works cannot be too vigorously encouraged in the interest of hygiene and salubrity.

From the foregoing it is perfectly evident that the inter-oceanic canal itself, together with the channels provided to regulate the flow of the rivers and streams on both sides of it, will thoroughly drain out and destroy the morasses, the depth of the excavations and dredging under water will entirely remove the causes of animal and vegetable effluvia and change the constitution of the land from a malarious to a salubrious one. The marshes once gone the transformation becomes permanent as there is always a subsoil drainage that would constantly be carried off by the channels as well as by the canal itself, which by the movement of the tide will keep up the circulation of the water—a point of the utmost hygienic value. The malignant fevers peculiar to these regions are singularly absent from the sea-coast proper, and the canal will convert the isthmus into a kind of sea-board, making its borders comparatively a continuance of the coast. The salt air which now loses its effect a few miles inland, would then follow the line of the canal from the Atlantic through to the Cu ebra Division to the Pacific, circulating freely and permeating with its beneficial influences the whole isthmus. It remains to consider briefly in what manner the atmospheric changes will be brought about and maintained.

The Atlantic and Pacific ocean contain a greater proportion of salt than most oceans and seas. The Atlantic is richer in salt in the eastern than in the western part, and both show their maximum per centage of salt a few degrees north of the equator, this per centage increasing with the depth as is easily explained. Sea water is more concentrated on the surface by evaporation; in the depth, on the other hand the specificgravity increases as the temperature decreases. The Atlantic and Pacific, however, are exceptions in this respect having their greatest per centage of salt near the surface,* which makes their vapors all the more desirable as disinfecting agents. Comparing the component parts of salt sea with those of fresh water it is evident that the ocean being the reservoir of what is drawn from the main land by fresh waters, contains all the component parts of the latter with greater quantities of salt, bittern, sulphate of lime, chlorides of magnesium and sodium with traces of copper, lead, zinc and other metals. From the chlorides

* Knop, Agricultur Chemie.

combined with the metallic ingredients, sea water derives its purifying properties. Forchhammer, in order to make the proportion scientifically correct, reduced the sum of all the solid component parts that sea water leaves by evaporation to a unit as to the quantity of chlorine. The proportionate number obtained when the whole sum of solid component parts is divided by the quantity of chlorine contained therein, he named: the "middle co-efficient for sea water." The middle co-efficient for sea water of the world being, according to him, 1,811; the minimum 1,806, the maximum, 1,814; a few degrees north of the equator, 6°–10° N. latitude; consequently, its power of disinfecting is greatest just opposite the isthmus on both the Atlantic and Pacific sides.

Fresh water always contains a somewhat smaller amount of dissolved contents than sea water or water from salt springs. This is partly the result of dilution under the direct influence of rain or some other form of meteoric water and partly due to chemical process, when the springs feeding rivers come into contact with the atmospheric air. The amount of dissolved organic substances in fresh water is, nevertheless, very large. This is especially the case in the Chagres, Obispo and Trinidad rivers. These substances, a prolific source of miasma, carried into the rivers are diffused throughout the running water containing also dissolved oxygen, exposed to whose action they are gradually decomposed and converted into their final products, chief among which are water, ammonia, the nitrites and nitrates. The dissolved oxygen playing under the most favorable circumstances an insignificant part in the natural purification of rivers, falls entirely out of mind when it comes to the prevention of putrefaction or of accumulation of organic matter in such as these; into whose courses putrescent organic substances are constantly carried as sewage from the hamlets and numerous huts along their banks, as well as by the natural surface drainage, in quantities sufficient to annul any chemical process of oxygen in their waters. In addition, they contain mechanically suspended substances the amount of which varies according to the rainfall. The liability of putrescent organic matter in fresh water to be decomposed and form ammonia by evaporation during the dry season, must be taken as a test of the quality of the water in so far as such matter is concerned and as a measure of the amount it contains. The presence of the nitrites and nitrates being not only injurious in itself but what is far more important indicates either sewage contamination or other extraneous matter, whether, from stagnant malarial pools or morasses ; they indicate this as they themselves are the products of nitrogeneous organic material. Decomposition being incomplete, these rivers and streams are filled with organic substance in its most objectionable condition, the vapors arising from their surfaces being impregnated with poisonous matter accordingly.

From the facts already stated, it will be possible to form some idea of the geological importance attaching to the constant circulation of water from ocean to ocean, as well as to the atmospheric changes derived by evaporation from the surface of the canal. As mentioned it is to the chlorides combined with the metals that sea water owes its purifying and disinfecting qualities. The chlorides of zinc and lead, in particular, both possess antiseptic and deodorizing properties in a combined watery solution o 1,100. If a given quantity of fresh water from a stagnant slimy pool be placed in a test glass, an equal quantity in another, and to the first be added a small proportion of this solution, to the other, nothing, it will be observed at the end of 24 hours, that the first glass is entirely free from those parasites whose presence marks decomposition, in the second will be seen vibrios, leptothrixe and ciciliated infusoria. Not only this, but the power of these two chlorides alone, the first as an antiseptic the second as a deodorizer,is so great as to overcome the decomposition of excreta and to render pure the air of sick wards, where they are frequently employed in the large hospitals of London. The influence of the chlorides of lime and soda, upon foul atmosphere, is too well known to need further notice. Such being the properties of the chlorides, they would be at least as active, in their natural state suspended in the air, in annihilating any organic germs contained in vapor arising from the miasmatic rivers, against which their work will be chiefly directed as the marshes, the principal trouble, will have ceased to exist when the canal is finished.

When once the deep cut is made, and this immense volume of strongly impregnated salt water shall pass through the isthmus, the purifying salt sea air will roll along with it, and drive out the malarious atmosphere that now hovers over it. The atmospheric circulation will be constant and with the grand system of drainage inaugurated, the whole country will be converted into a healthy one, the average of human life raised, and the prolific source of infectious fevers abolished.*

*This state of things fully established, will be perpetual, as there are no earthquakes at †Panama, which cannot be said of the volcanic regions of Nicaragua.

†Berghaus, Atlas, Dresden.

Those who will profit first are the now unfortunate inhabitants of that locality. Even in this generation the salubrity that the country will derive from the inter-oceanic canal may permit them to see their children growing up strong, healthy, and well started in life. The government of Colombia will be under double obligation to the great genius who brings new commercial life and health to its borders.

The maritime gain in relation to public hygiene, will be of no less importance to foreign nations. The chances of carrying infectious diseases to foreign ports by sailors who have been on shore, by goods or ships, will be reduced to nul, when vessels without touching at Colon or Panama, pass through the canal. Almost every epidemic of Asiatic cholera and yellow fever, has been traced directly to causes such as these, it having been conclusively demonstrated that the disease was propagated from infected districts.

Conversely the inhabitants of Colombia would here again profit as vessels having the germs of infectious diseases on board, would continue on their course, thus avoiding intercourse with the shore. The United States have so often been afflicted by scourges such as these, traceable directly to this means of propagation, that the high purpose of aiding to prevent their importation, should be sufficient to induce them to co-operate in such a project.

In the recent report of the American Public Health Association, the commission on yellow fever presented some valuable papers on this point, of which the following is an extract: "The great object to be aimed at is to prevent the germs or fomites of this dreadful pestilence from having access to our people. The only certain and sure preventative of yellow fever is absolute non-intercourse with the parts where yellow fever is indigenous; further, in respect to the various towns which we visited, and which were points of epidemic prevalence, the testimony showing its importation was direct and convincing in its character. The transmission of yellow fever between points separated by any considerable distance, appeared to be wholly due to human intercourse. In some instances the poison was carried in clothing or about the persons of people going from infected districts. In other instances it was conveyed in such fomites as cotton bagging or goods of some description, bedding and blankets, etc."

There is always a possibility of conveying the poison by means of goods sent by rail or by the personal efferts of men. That is to say, human beings may possess no susceptibility to yellow fever, may even remain healthy and yet the poison of the disease conveyed in clothing, personal effects, cargo or bilge water may have retained its capacity for infection. Touching the terrestrial circumstances it is particularly noticeable that the disease is chiefly in cities which have maritime commerce, whether they be upon the sea-coast or important rivers. Here it is almost invariably that quarter of the city lying nearest the harbor, that is the first to be attacked in every new epidemic, and though all the unfavorable conditions may have been developed on board in the highest degree; yellow fever has never yet been on a ship that has not in some way come into communication with the land or with some other ship where the disease already prevailed. The acknowledged cause of yellow fever being a living miasm which has hitherto entirely eluded microscopic demonstration, but the existence of which is argued from very many facts.

In the lines immediately preceding, use has been made of the valuable article on yellow fever, published in Ziemssen's Cyclopædia, by Dr. Fritz Haenisch, medical officer of the German navy. In that capacity he had abundant opportunity to study this disease whilst visiting different ports in the Mediterranean, Madeira, the West Indies, North and South America, the Azores and Portugal. This living miasm is held suspended in the air emanating from salt and fresh marshes, and may be carried many miles by the currents of air and wind; a source of propagation of no less import being land and maritime intercourse. And this theory so universally indorsed by science is equally applicable to Asiatic cholera, the pest, typho-malarial fever and to infectious diseases as a whole class. The relation of railway traffic, commerce, goods, passengers, etc., to the spread infectious diseases by land though direct, are no less intimate than those of maritime intercourse; it having been shown by reports from which might be quoted hundreds of instances, that in every case these diseases were imported either by the ships themselves or by persons manning them. At present, merchandise in transit through the isthmus of Panama has to be landed, stored, transported by rail, stored again and lightered. Even if this be effected with the utmost possible despatch, considerable delay must of necessity be experienced. Long enough at shortest for bales, boxes, hides, etc., to receive and carry the germs of disease, either from the stevedores, trackmen and workmen handling them, or from atmospheric influences. It is easy to see the benefit when vessels, coming either from the Atlantic or Pacific, able to cross from one ocean to the other without delay and within one day, will thus aid materially in preventing the spread of infectious disease to all parts of the world.

The people of the United States who are pre-eminently interested are invited to join in this great work and aid M. De Lesseps to dredge the marshes, to pierce the mountain and mingle the waters of the Atlantic with those of the Pacific. The inter-oceanic canal will bring to commerce a prosperity greater than it has yet attained; it will give health and life to a people at home; reduce the dissemination of death and disease abroad, and be a blessing to humanity throughout ages to come.

